# Predicting binary, discrete and continued lncRNA-disease associations via a unified framework based on graph regression

**DOI:** 10.1186/s12920-017-0305-y

**Published:** 2017-12-21

**Authors:** Jian-Yu Shi, Hua Huang, Yan-Ning Zhang, Yu-Xi Long, Siu-Ming Yiu

**Affiliations:** 1 School of Life Sciences, Northwestern Polytechnical University, Xi’an, 710072 China; 2 School of Software and Microelectronics, Northwestern Polytechnical University, Xi’an, 710072 China; 3 School of Computer Science, Northwestern Polytechnical University, Xi’an, 710072 China; 4 Department of Computer Science, the University of Hong Kong, Hong Kong, 999077 China

**Keywords:** lncRNA-disease association, Graph regression, Prediction, Discrete, Continued, Sequence feature, Semantic similarity

## Abstract

**Background:**

In human genomes, long non-coding RNAs (lncRNAs) have attracted more and more attention because their dysfunctions are involved in many diseases. However, the associations between lncRNAs and diseases (LDA) still remain unknown in most cases. While identifying disease-related lncRNAs in vivo is costly, computational approaches are promising to not only accelerate the possible identification of associations but also provide clues on the underlying mechanism of various lncRNA-caused diseases. Former computational approaches usually only focus on predicting new associations between lncRNAs having known associations with diseases and other lncRNA-associated diseases. They also only work on binary lncRNA-disease associations (whether the pair has an association or not), which cannot reflect and reveal other biological facts, such as the number of proteins involved in LDA or how strong the association is (i.e., the intensity of LDA).

**Results:**

To address abovementioned issues, we propose a graph regression-based unified framework (GRUF). In particular, our method can work on lncRNAs, which have no previously known disease association and diseases that have no known association with any lncRNAs. Also, instead of only a binary answer for the association, our method tries to uncover more biological relationship between a pair of lncRNA and disease, which may provide better clues for researchers. We compared GRUF with three state-of-the-art approaches and demonstrated the superiority of GRUF, which achieves 5%~16% improvement in terms of the area under the receiver operating characteristic curve (AUC). GRUF also provides a predicted confidence score for the predicted LDA, which reveals the significant correlation between the score and the number of RNA-Binding Proteins involved in LDAs. Lastly, three out of top-5 LDA candidates generated by GRUF in novel prediction are verified indirectly by medical literature and known biological facts.

**Conclusions:**

The proposed GRUF has two advantages over existing approaches. Firstly, it can be used to work on lncRNAs that have no known disease association and diseases that have no known association with any lncRNAs. Secondly, instead of providing a binary answer (with or without association), GRUF works for both discrete and continued LDA, which help revealing the pathological implications between lncRNAs and diseases.

## Background

According to the central dogma of molecular biology, DNAs should be transcribed into different kinds of RNAs [1]. The transcriptional outputs of DNAs comprise both protein-coding messenger RNAs (mRNAs) and non-coding RNAs (ncRNAs). The latter was commonly regarded as transcriptional noise [1]. However, the Human Genome Project unexpectedly reveals that only ~2% of chemical bases in the genome sequence were transcribed into mRNAs [1], while the remaining bases accounting for a very big portion of the whole genome are transcribed to ncRNAs [2]. As a result, ‘Why is the majority of the genome non-coding?’ becomes one of the core questions in genomics [3].

In recent years, biological experiments show the critical biological roles of ncRNAs, which are involved in regulation of transcription, translation, RNA modification, maturation or transportation and in epigenetic modification of chromatin structures [3]. ncRNAs have amazing variety in structure and in gene regulation outcomes. As the number of known functional ncRNAs is increasing [4], various RNA species in the human genome can be roughly categorized as short (sncRNAs) and long (lncRNAs) groups by sequence length (200 nucleotides generally). sncRNAs, such as siRNA (small inhibitory RNA), miRNA (microRNA), piRNA (piwi RNA) and antisense RNA, have less than 200 nucleotides (nts) and are highly conserved in different species and have a key role in transcriptional and post-transcriptional silencing of genes. On the other hand, lncRNAs with lengths of over 200 nts, are poorly conserved and have low expression levels and high tissue specificity. lncRNAs are usually encoded as intergenic, intronic or overlapping regions [5], unfortunately, how they perform their diverse functions is still largely unknown [6, 7].

The dysfunction (e.g. mutations and de-regulations [8, 9]) of lncRNAs is heavily involved in the development or progression of diseases, such as cardiovascular disease [10] and cancer [11]. Thus, lncRNAs could be novel molecules for disease diagnosis and therapy [3, 9, 12]. Nevertheless, the number of lncRNAs, which has been functionally characterized and associated with diseases, is extremely small. The relationship between lncRNAs and human diseases remains unknown in most cases up to now. Consequently, there is an increasing need to identify lncRNAs-disease associations (LDA) on a genome-wide scale [12].

However, identifying disease-related lncRNAs based on biological experiments is still a great challenge because of the lengthy process (time) and high cost. Computational approaches provide alternatives for identifying possible lncRNA-disease associations for further study and validation in wet lab [13]. Besides, computational approaches can also help provide clues on the underlying mechanism of various lncRNA-caused diseases and accelerate the identification of potential biomarkers for disease diagnosis, treatment, prognosis and prevention [3, 14].

Computational approaches, especially based on machine learning, such as Laplacian Regularized Least Squares [15], network topology inference [16] [17], Random Walk [13, 18] and SVM [19], have been developed to predict potential LDA, based on the assumption that similar diseases tend to be associated with similar lncRNAs in function [19].

Most of the former approaches only focus on the predicting scenario between the lncRNAs with known associating diseases and the diseases with known associating lncRNAs. However, the majority of lncRNAs has no known disease association. Also, there exist more and more diseases which have no known association with any lncRNAs. It is desirable to have an approach that can work on these lncRNAs and diseases. Moreover, to the best of our knowledge, existing computational approaches only work on binary LDA (i.e. only reports if there is an association or not), which cannot reflect and reveal many biological facts or knowledge. For example, a disease-associated lncRNA may cause the disease by dysregulating diverse proteins [3, 20]. Binary associations cannot show the number of proteins involved in the associations as well as the intensity of the associations.

To address abovementioned issues, we propose a Graph Regression-based approach which provides a Unified Framework (GRUF) for four predicting tasks, including the traditional task solved by existing approaches that work on lncRNA with known disease association and diseases having known association with some lncRNAs. GRUF is also able to work for lncRNAs with no known disease association and diseases without known association with any lncRNAs. Moreover, instead of predicting binary LDA only, GRUF can also work for both discrete and continued LDA, which helps to reveal the implications between lncRNA and pathology. We demonstrate the superiority of GRUF by both the comparison with three state-of-the-art approaches and the comprehensive prediction across distinct tasks over multi-type associations. In addition, its effectiveness is further verified by validating the prediction of novel lncRNA-disease associations from both medical literature and a related database.

## Methods

### Problem formulation

**Fig. 1:**
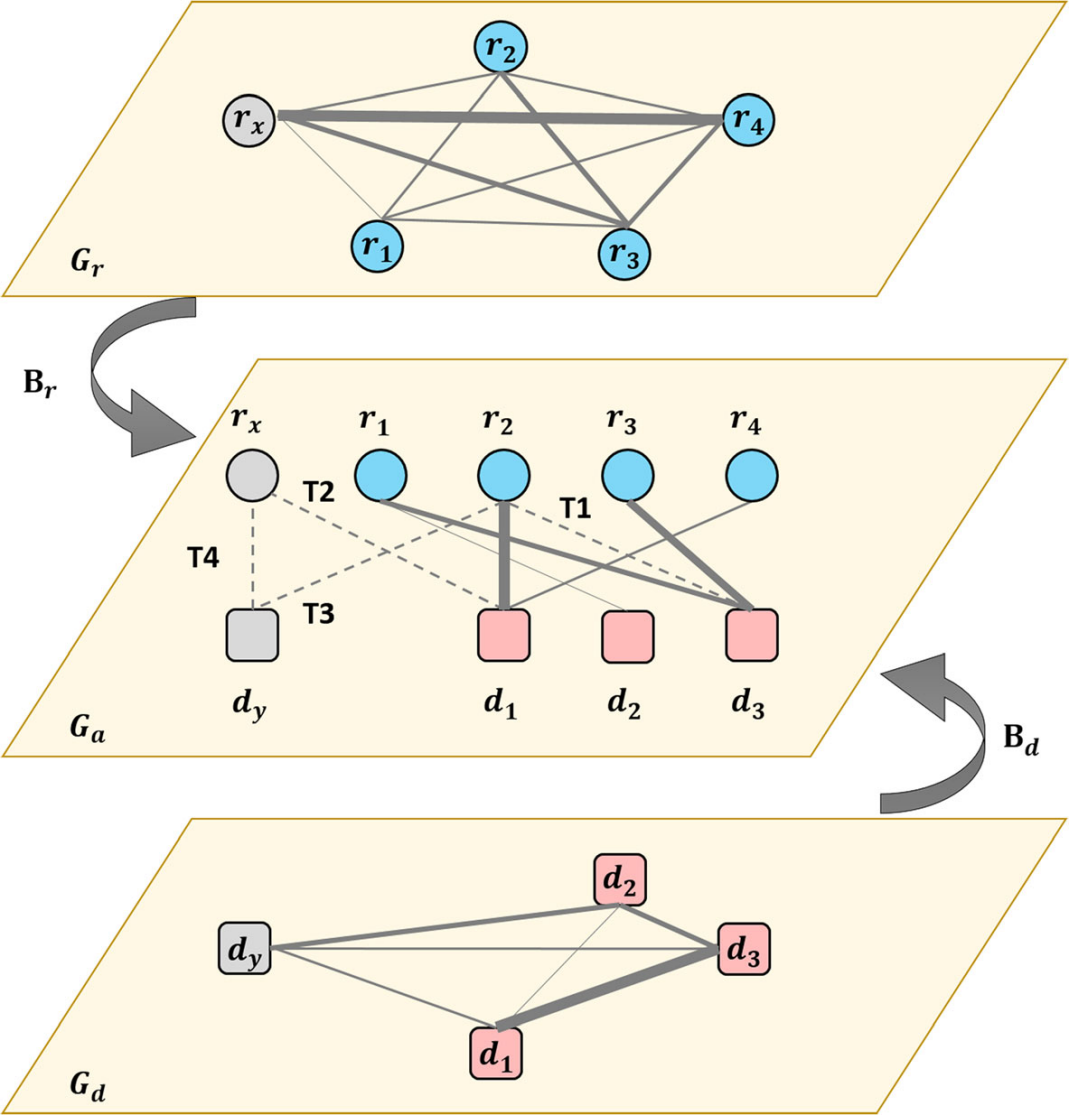
Graph regression for predicting the associations between lncRNAs and diseases. From top to bottom, *G*_*r*_, *G*_*a*_ and *G*_*d*_ are listed. Circle nodes and rounded square nodes denote lncRNAs and diseases respectively. In *G*_*r*_ and *G*_*d*_, lines denote the similarities between nodes. In *G*_*a*_, solid lines linking nodes represent LDAs and dashed lines denote the pairs to be predicted. T1, T2, T3 and T4 account for four predicting tasks

Given a set of associations between *m* known lncRNAs {*r*_*i*_} (denoted as **R**) and *n* known diseases {*d*_*j*_} (denoted as **D**), we have four predicting scenarios/tasks (Fig. 1). The first one (T1) is the traditional one handled by existing approaches, which infers how likely there are novel associations between **R** and **D**, where both **R** and **D** have other known associations. The second one (T2) is to find potential associated diseases from **D** for an lncRNA *r*_*x*_, which has no known disease association. Symmetrically, the third one (T3) is to find potential associated lncRNAs from **R** for a disease *d*_*y*_, with no known association with any lncRNAs. The last one (T4) is the most difficult task which deduces how likely there are potential associations between lncRNAs with no known disease association and diseases with no known association with any lncRNAs. Solving T4 could provide clues for researchers to further investigate unexpected associations between lncRNAs and diseases. Moreover, lncRNAs without known disease association and diseases without known association with lncRNAs are the majority.

The set of known LDAs between **R** and **D** can be organized into an association matrix **A**_*m×n*_. We consider three types of associations between lncRNAs and diseases, including binary, discrete and continued LDAs. The corresponding association matrices are denoted as ${\mathbf{A}}_{m\times n}^b$, ${\mathbf{A}}_{m\times n}^d$ and ${\mathbf{A}}_{m\times n}^c$. Traditionally, in ${\mathbf{A}}_{m\times n}^b$, *a* ^*b*^(*i*, *j*) = 1 if there is a known interaction between lncRNA *r*_*i*_ and disease *d*_*j*_, and *a* ^*b*^(*i*, *j*) = 0 otherwise. By contrast, in ${\mathbf{A}}_{m\times n}^d$, *a* ^*d*^(*i*, *j*) ∈ *ℕ* ^+^ (positive integers) if there is a known interaction between lncRNA *r*_*i*_ and disease *d*_*j*_, and *a* ^*d*^(*i*, *j*) = 0 otherwise. In ${\mathbf{A}}_{m\times n}^c$, *a* ^*c*^(*i*, *j*) ∈ *ℝ* ^+^ (positive real numbers) and, *a* ^*c*^(*i*, *j*) ≥ 1 if there is a known interaction between lncRNA *r*_*i*_ and disease *d*_*j*_, and *a* ^*c*^(*i*, *j*) < 1 otherwise. ${\mathbf{A}}_{m\times n}^d$ is able to provide more information than ${\mathbf{A}}_{m\times n}^b$, such as the number of proteins (or its coding genes) involved in the associations, while ${\mathbf{A}}_{m\times n}^c$ can further reflect how strong the association is (i.e., the intensity of LDA). Three kinds of associations can be represented as a binary graph, a weighted graph and a completed weighted graph respectively, in which lncRNAs and disease are nodes and their associations are edges. For short, the graph is denoted as *G*_*a*_.

We aim to develop a unified framework for predicting LDAs in the above four scenarios. Involving new nodes, the prediction in either T2, T3 or T4 can be regarded as a cold-start problem in recommendation systems. Except for the topology of association graph, additional attributes of nodes should be integrated in T2, T3 and T4, which have a requirement of predicting links for nodes having no existing links at all.

Note that pairwise lncRNA similarities can be independently measured with respect to the topology of LDA graph, and are organized into a lncRNA similarity graph *G*_*r*_. Similarly, pairwise disease similarities can be organized into a disease similarity graph *G*_*d*_. Their symmetric adjacent matrices, represented as **S**_**r**_ and **S**_**d**_ respectively, are further integrated with **A**_*m×n*_ to perform the prediction of LDAs.

### Graph regression

**Fig. 2:**
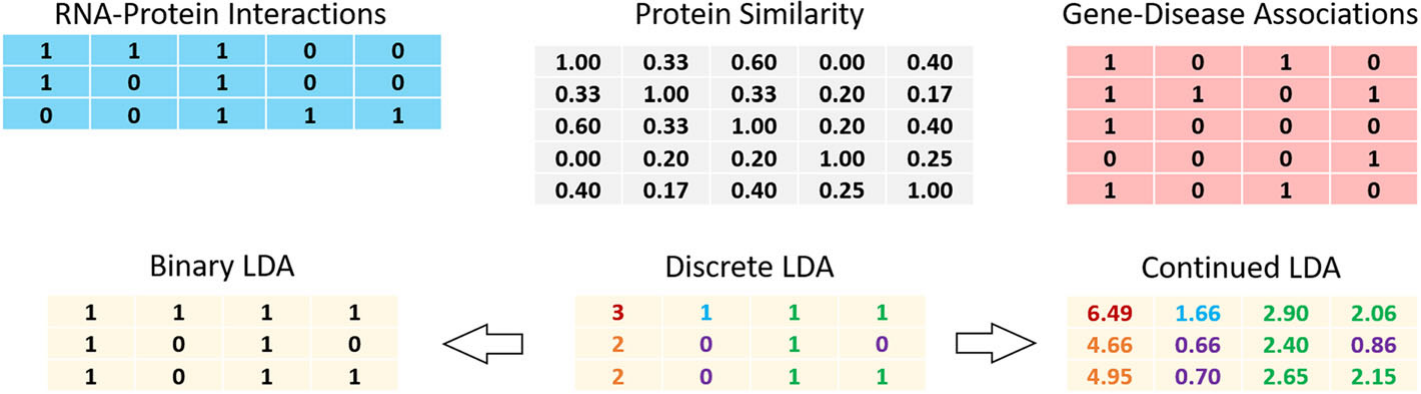
A toy example of illustrating three kinds of LDAs involving 3 lncRNAs and 4 diseases. The first row shows a 3×5 binary lncRNA-protein interaction matrix, a 5×5 protein similarity matrix and a 5×4 binary gene-disease association matrix from left to right. The second row lists three kinds of 3×4 LDA matrices, including a binary matrix, a discrete matrix, and a continued matrix. The entries highlighted by different colors in discrete and continued matrices have different values. Binary LDA provides a coarse information about how a lncRNA is associated with a disease, while discrete LDA and continue LDA provide the number of proteins involved in LDA and the intensity of LDA for that question respectively

We transform the predicting task into a graph regression between *G*_*r*_, *G*_*d*_ and *G*_*a*_ (Fig. 2). The graph regression is synchronously performed in three latent spaces, associating space, lncRNA similarity space and disease similarity space. Therefore, the graph regression can be formulized as follows,

1 $$ \left\{{\mathbf{A}}_{\mathbf{r}}^{\ast },{\mathbf{A}}_{\mathbf{d}}^{\ast },{\mathbf{F}}_{\mathbf{r}}^{\ast },{\mathbf{F}}_{\mathbf{d}}^{\ast },{\mathbf{B}}_{\mathbf{r}}^{\ast },{\mathbf{B}}_{\mathbf{d}}^{\ast}\right\}=\arg \min \kern1em \parallel \mathbf{A}-{\mathbf{A}}_{\mathbf{r}}{\mathbf{A}}_{\mathbf{d}}^T{\parallel}_F^2+\parallel {\mathbf{S}}_{\mathbf{r}}-{\mathbf{F}}_{\mathbf{r}}{\mathbf{F}}_{\mathbf{r}}^T{\parallel}_F^2+\parallel {\mathbf{S}}_{\mathbf{d}}-{\mathbf{F}}_{\mathbf{d}}{\mathbf{F}}_{\mathbf{d}}^T{\parallel}_F^2+\parallel {\mathbf{A}}_{\mathbf{r}}-{\mathbf{F}}_{\mathbf{r}}{\mathbf{B}}_{\mathbf{r}}{\parallel}_F^2+\parallel {\mathbf{A}}_{\mathbf{d}}-{\mathbf{F}}_{\mathbf{d}}{\mathbf{B}}_{\mathbf{d}}{\parallel}_F^2. $$

The first three items in the above objective function account for three low-rank decompositions, which map *G*_*a*_ into the associating space, *G*_*r*_ into the lncRNA similarity space and *G*_*d*_ into the disease similarity space respectively. While the last two items account for the regression between the associating space and the lncRNA similarity space, and the regression between the associating space and the disease similarity space. For elegance, the regularization items are omitted. In details, the lncRNAs and diseases in *G*_*a*_ are mapped into an *m* × *r* lncRNA associating matrix **A**_**r**_ (RAM) and a *n* × *r* disease associating matrix **A**_**d**_ (DAM); the lncRNAs in *G*_*r*_ are mapped into an *m* × *p* lncRNA latent feature matrix **F**_**r**_ (RLFM); the diseases in *G*_*d*_ are mapped into an *n* × *q* lncRNA latent feature matrix **F**_**d**_ (DLFM); the *p* × *r* matrix **B**_**r**_ and the *q* × *r* matrix **B**_**d**_ are the corresponding regression coefficient matrices.

When assuming that the five items in formula (1) are independent, we give a simple solution for above optimization problem by minimizing the items individually. For the low-rank decompositions, we apply Singular Value Decomposition (SVD) to generate RAM, DAM, RLFM and DLFM respectively by $\mathbf{M}\overset{SVD}{=}{\mathbf{U}\boldsymbol{\Sigma } \mathbf{V}}^T=\left(\mathbf{U}\sqrt{\boldsymbol{\Sigma}}\right){\left(\mathbf{V}\sqrt{\boldsymbol{\Sigma}}\right)}^T={\mathbf{LR}}^T$, where **M** denotes **A**, **S**_**r**_ or **S**_**d**_. For the regression, we utilize Partial Least-Squares (PLS) regression to generate **B**_**r**_ and **B**_**d**_ individually.

Sequentially, the proposed graph regression model enables us to solve T1, T2, T3 and T4 in a unified framework. The predicted confidence scores of being a potential LDA in all the tasks are defined as

2 $$ {\tilde{\mathbf{A}}}_{\left(\mathrm{T}1\right)}={\mathbf{F}}_{\mathbf{r}}{\boldsymbol{\Theta} \mathbf{F}}_{\mathbf{d}}^T,\kern1em {\tilde{\mathbf{A}}}_{\left(\mathrm{T}2\right)}={\mathbf{F}}_{\mathbf{r},x}{\boldsymbol{\Theta} \mathbf{F}}_{\mathbf{d}}^T,\kern1em {\tilde{\mathbf{A}}}_{\left(\mathrm{T}3\right)}={\mathbf{F}}_{\mathbf{r}}{\boldsymbol{\Theta} \mathbf{F}}_{\mathbf{d},y}^T,\kern1em {\tilde{\mathbf{A}}}_{\left(\mathrm{T}4\right)}={\mathbf{F}}_{\mathbf{r},x}{\boldsymbol{\Theta} \mathbf{F}}_{\mathbf{d},y}^T, $$

where **F**_**r**, *x*_, calculated from the lncRNA similarity matrix, is the latent feature vectors of newly given lncRNAs *r*_*x*_ (having no association with diseases), **F**_**d**, *y*_, calculated from the disease similarity matrix, is the feature vectors of newly given diseases *d*_*y*_ (having no association with lncRNAs), and $\boldsymbol{\Theta} ={\mathbf{B}}_{\mathbf{r}}{\mathbf{B}}_{\mathbf{d}}^T$ is the bi-regression coefficient matrix, calculated by the known lncRNA set **R** and the known disease set **D**.

Moreover, this framework is flexible when there are no similarity graph available but real-world feature vectors, such as lncRNA sequence features. In this case, **Θ** builds the bridge between the features of lncRNAs, the features of diseases as well as the associations between them. Its entries indicate the importance of the pairs between lncRNA features and disease features among associations and non-associations. Compared with latent features, real-world features are usually able to provide more straightforward elucidation of why an lncRNA is associated with a disease.

### Generation of non-binary association

Considering the discovery that the interaction between lncRNAs and RNA-binding proteins (RBPs) can reveal the roles of lncRNAs in the multi-layered transcriptional [21] and the possible involvement in the alterations of cellular pathways [3], we hope RBPs may contribute to better lncRNA annotations in understanding disease-related regulations. In addition, the genes coding RBPs are probably associated with diseases. Therefore, we utilized both lncRNA-RBP interactions and gene-disease associations to construct discrete and continued lncRNA-disease associations, which are reflected by integer and real values respectively but not binary indicators. For convenient description, the terms of gene and protein refer to as the same object in the following texts.

The traditional binary association ${\mathbf{A}}_{m\times n}^b$ can be easily generated by checking whether or not an lncRNA and a disease share common genes/proteins. If yes, they are associated with each other. The discrete association ${\mathbf{A}}_{m\times n}^b$ can be generated by counting the number of common genes/proteins. The numbers account for the values of discrete associations. The continued association is generated as follows. Let **A**_*r* − *p*_ be the interaction matrix between lncRNAs and RBPs, **A**_*d* − *g*_ be the association matrix between diseases and disease-related genes, and **S**_*P*_ is the symmetric similarity matrix between the proteins, which are coded by the common genes between the coding genes of RBPs in **S**_*r* − *p*_ and the disease-related genes in **A**_*d* − *g*_. We believe that **the larger the number of common genes/proteins is and the more similar they are, the more possible the RNA is associated with the disease**. Thus, the continued association matrix between lncRNAs and diseases can be defined as ${\mathbf{A}}_{m\times n}^c={\mathbf{A}}_{r-p}{\mathbf{S}}_P{\mathbf{A}}_{d-g}$.

A toy example illustrates these three types of LDAs in Fig. 2. Three observations can be drawn: (1) the binary LDA matrix only denotes whether or not lncRNAs are associated with diseases; (2) beyond the binary matrix, the discrete matrix indicates an extra information of how many RNA binding proteins or their coding genes are involved in each LDA; (3) going deeper, the continued matrix shows the intensity of LDAs, which can distinguish the entries even having the same number of RBPs (e.g. the blue entry and the green entries). As a result, compared with the binary associations, the union of the discrete associations and the continued associations provides evidence for functionally annotating the roles of lncRNAs and discovering their underlying mechanisms associated with diseases.

### Similarity measurement

The similarity matrices of lncRNA, protein and disease are constructed as follows. First, the occurring frequency of K-mer, a short substring consisting of K letters derived from the set {*A*, *C*, *G*, *U*} is applied to characterize an RNA sequence. In general, the occurring frequency of 4-mer is applied to calculate the sequence features of lncRNA [22]. Considering that the binding between RNAs and proteins occurs on local zones in sequence, we enhance the original 4-mer feature by dividing a sequence of lncRNA into multiple (e.g. 35) sequence segments of approximately same lengths, calculating 4-mer features separately and concatenating them into one feature vector. Then, the pairwise similarity between any two lncRNA sequences, accounting for an edge in lncRNA graph *G*_*r*_, can be generated from their feature vectors **r**_*i*_ and **r**_*j*_ by 1/(1 + *dist*(**r**_*i*_, **r**_*j*_)), where *dist* denotes Euclidian distance.

Secondly, considering the importance of specific properties of amino acids in diverse kinds of bindings, we adopted the approach in [23] to calculate protein sequence similarity as follows. According to both dipole moments and side chain volume, twenty kinds of amino acids are firstly separated into 7 groups, {*A*, *G*, *V*}, {*I*, *L*, *F*, *P*}, {*Y*, *M*, *T*, *S*}, {*H*, *N*, *Q*, *W*}, {*R*, *K*}, {*D*, *E*}, and {*C*}. Then, protein sequences are encoded into a new type of sequences, which consists 7 corresponding letters with respect to those groups. Last, the occurring frequency of 3-mer is applied on these encoded sequences to represent protein sequences. The pairwise similarity between two protein sequences, which feature vectors are represented as **p**_*i*_ and **p**_*j*_ respectively, can be defined by 1/(1 + *dist*(**p**_*i*_, **p**_*j*_)) as well.

Thirdly, we calculated disease similarity with the help of MeSH, which provides a hierarchical disease classification system containing a set of semantic disease descriptors (nodes) [24]. Each descriptor accounts for a disease category containing one or more diseases. Meanwhile, a disease may be assigned to one or more categories. For example, *Breast Neoplasms* belongs to two categories, *C04.588.180* and *C17.800.90.500*. Base on MeSH descriptors, each disease can be represented a directed acyclic graph (DAG) and the pairwise similarity of two diseases is calculated by comparing their DAGs. The more the common parts of their DAGs are, the more similar they are. The details can be found in [25]. We adopt this semantic similarity as the disease similarity when predicting lncRNA-disease associations.

### Assessment

The assessment of a predicting approach should consider two crucial factors, including algorithm validation and performance evaluation. Algorithm validation is always implemented by the well-known Cross Validation (CV). Remarkably, when assessing approaches, the appropriate schemes of CV for different scenarios should be adopted, otherwise over-optimistic results are perhaps obtained [26, 27]. We generated different tasks of CV under four scenarios as follows (see also Fig. 1):

- CV_T1: CV performed on lncRNA-disease pairs, where random entries (lncRNA-disease pairs) in A were selected for testing and the remaining entries were used for training.
- CV_T2: CV performed on lncRNAs, where random rows corresponding to lncRNAs in A were blinded for testing and the remaining rows were used for training.
- CV_T3: CV performed on diseases, where random columns in A (accounting for diseases) were blinded for testing and the remaining columns were used for training.
- CV_T4: CV performed on lncRNA-disease pairs, where random entries in A were selected for testing, but all the rows and columns containing the testing entries should be blinded for testing as well as training simultaneously. In other words, both the rows and the columns in A for training contain NONE of the testing entries.

We adopt K-fold cross validation (K-CV) to assess our approach on different predicting scenarios. The objects in an LDA matrix are randomly split into K subsets with approximately equal sizes. In each round of CV, one subset of objects is taken as the testing set while the union of other subsets of objects is taken as the training set. This procedure keeps running K-1 rounds by assigning each subset of objects as the testing set in turn. Here, the term ‘object’ refers to as the entries of LDA in CV_T1 and CV_T4, while as the rows and the columns of LDA in CV_T2 and CV_T3.

Moreover, over these CV schemes, we use two metrics to evaluate the performance of LDA prediction. One is the popular Area Under the receiver operating characteristic Curve (AUC), which can be calculated according to the predicted confidence scores of positive and negative entries. In the binary prediction of LDA, known LDAs and other lncRNA-disease pairs are assigned with positive labels and negative labels respectively. AUC is appropriate to binary LDA prediction, however, is unavailable to discrete and continued prediction. We design a strategy to accommodate AUC for them.

Because there is a one-to-one correspondence between each entry of binary LDA matrix(BAM) and its enriched entry in either discrete LDA matrix (DAM) or continued LDA matrix (CAM), the binary values of the entries in BAM can be taken as the binary labels of those entries in DAM or CAM. Once the labels are set, the predicted confidence scores generated by discrete prediction or continued prediction can be used to calculate AUC by the same way as that in binary prediction.

However, AUC is not enough to measure the performance of discrete prediction or continued prediction because it can only indicate how well the approach can distinguish LDA from non-LDA. Therefore, another metric, *Correlation*, is proposed as an enhanced measure of discrete prediction or continued prediction. It indicates the consistency between the intensity of LDA and its predicted confidence scores. The higher, the better. A perfect predicting model generates the predicted scores, which are completely correlated with DAM or CAM.

## Result and discussion

### Datasets

We collected three datasets to evaluate GRUF. The first, denoted as DB1, was used as a benchmark dataset in former approaches [15, 28] and was also used to build a web server of predicting binary lncRNA-disease association in the most recent work [19]. DB1 contains 117 lncRNA, 159 diseases, and 285 binary associations between them. It also contains two lncRNA similarity matrices (sequence similarity and disease association-based similarity) as well as five disease similarity matrices (gene functional similarity, GO-based similarity, PPI topology-based similarity, PPI’s shorted path-based similarity and lncRNA association-based similarity). The lncRNA similarity matrices and the disease similarity matrices are combined respectively [19].

The second benchmark dataset, denoted as DB2, was collected from the recently published database, LncRNA2Cancer [29], which provides comprehensive experimentally supported associations between lncRNA and human cancer. After removing the lncRNAs having no available sequence in LncRNA2Cancer [29] and their associated cancers, we obtained 345 lncRNA, 93 cancers, and 747 binary associations between them in DB2. Using the approach in Section “Similarity Measurement”, we calculated the sequence similarity of RNA. Since LncRNA2Cancer contains no MeSH code for cancer, but the labels of International Classification of Diseases (ICD). We simply calculated the disease similarity of cancers by setting the pairwise disease similarity as 1 if two cancers belong to the same category in ICD, and 0 otherwise.

Moreover, we built the third dataset (DB3) to demonstrate the capability of GRUF in four kinds of predicting scenarios over three types of lncRNA-disease associations. In order to construct three kinds of LDAs, we collected the interactions between lncRNAs and their RBPs from LncRNADisease [30] and collected disease-associated genes and their diseases from DisGeNET [31]. We only kept the intersection of the coding genes of the proteins and the disease-associated genes, and selected their related lncRNAs and diseases respectively. Finally, DB3 contains 89 lncRNAs, 108 genes, and 406 diseases. In total, there are 154 experimentally supported lncRNA-protein interactions and 884 experimentally confirmed gene-disease associations.

When calculating lncRNA similarity, we split lncRNA sequences into 35 segments and obtained 8960-dimensional (35^∗^4^4^ = 8960) feature vectors based on 4-mer. Because all the values of 4-mer feature entries are small, we processed them by Z-score and obtained the normalized feature matrix, of which the columns have sample mean zero and sample standard deviation one. In addition, to accelerate the calculation of lncRNA similarity matrix, we performed Principal Component Analysis (PCA) on the feature vectors. After removing those dimensions, which have only entries of zeros within numerical accuracy, we obtained 88-d feature vectors. Moreover, we calculated the protein features for genes based on 7 amino acid groups, after turning gene sequences into protein sequences. Similarly, by PCA, we mapped the original 7^3^ -d feature vectors of proteins into 107-d feature vectors. After preprocessing lncRNA and protein feature vectors, we calculated the lncRNA similarity. The protein similarity was also calculated. The disease similarity was calculated directly based on MeSH descriptors of the diseases (see also Section Similarity Measurement).

### Comparison with state-of-the-art approaches

**Fig. 3:**
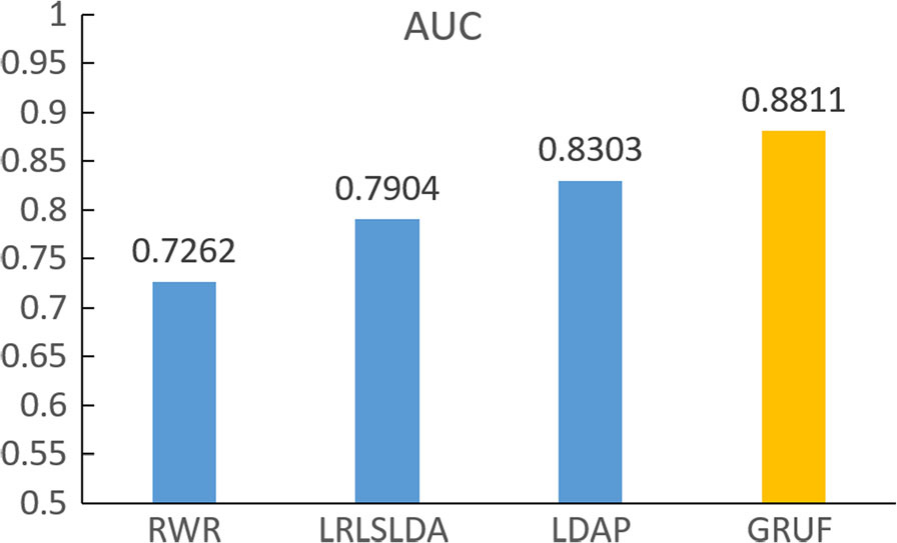
The comparison with three state-of-the-art approaches in the traditional scenario T1

In order to demonstrate the effectiveness of GRUF, we performed three experiments. We first compared our approach with three state-of-the-art approaches, RWR [28], LRLSLDA [15] and LDAP [19]. However, the former approaches are not designed to work in the case of non-binary lncRNA-disease associations and are also unable to meet the need of predicting associations for lncRNAs and diseases without known associations. The comparison was only performed in the case of predicting the traditional binary association in Scenario T1. To make a fair comparison, we adopted the same dataset (DB1), the same cross validation (leave-one-out), the same measure (AUC) as those in LDAP (the most recent approach). The result shows that our approach is significantly superior to those state-of-the-art approaches in terms of AUC (Fig. 3).

To our knowledge, there is no existing approach using DB2 as benchmark dataset since it was published very recently. Thus, we compared GRUF with two models, MLKNN [26] and RLS [27], which work on the similar form of problem (drug-target interaction prediction). As recommended in [26, 27], an extra metric, the area under precision-recall curve (AUPR), was adopted to measure the prediction performance with AUC together. Since those models were originally designed for Scenario T2 and T3, the prediction was run in the same scenarios under 10-CV (Table 1). The comparison of prediction shows that the performance of GRUF is significantly better than that of those models, especially in terms of AUPR.

**Table 1:** The comparison with three state-of-the-art models in Scenario T2 and T3

Scenario	Measure	MLKNN	RLS	GRUF
T2	AUC	0.8334	**0.8510**	0.8482
	AUPR	0.1064	0.1443	**0.1479**
T3	AUC	0.8377	0.5915	**0.8451**
	AUPR	0.1742	0.0971	**0.2442**

The best values are bold

### Predicting comprehensive lncRNA-disease associations

We demonstrated the prediction ability of our GRUF when encountering both discrete and continued association in three scenarios, T2, T3 and T4, which involve lncRNA and/or diseases with no known association. Ten-fold CV was run on DB3 to evaluate the performance of GRUF. In details, all lncRNAs and all diseases, with known associations, are randomly partitioned into 10 non-overlapping subsets of equal size respectively. In each round of the CV, each subset of lncRNAs is removed as the testing lncRNAs *Tst*_*r*_ and the remaining lncRNAs are referred to as the training lncRNAs *Trn*_*r*_ in T2. Similarly, each subset of diseases is removed as the testing diseases *Tst*_*d*_ and the remaining diseases are regarded as the training diseases *Trn*_*d*_, in T3. In T4, the sub-matrix containing all the entries between *Trn*_*r*_ and *Trn*_*d*_ in the association matrix A are labelled as the training part, only the submatrix containing the entries between *Tst*_*r*_ and *Tst*_*d*_ are labelled as the testing part, and the entries between *Tst*_*r*_ and *Trn*_*d*_ as well as those entries between *Trn*_*r*_ and *Tst*_*d*_ attend in neither training nor testing phases. Thus, T4 requires 10×10 cross validation. In addition, the results of predicting binary, discrete and continued association in T1 are listed for the comprehensive comparison.

Based on the predicted confidence scores that indicate how likely the testing lncRNA-disease pairs are potential LDA, we performed two investigations. The former examines how well GRUF separates LDA from non-LDA for binary, discrete, continued LDAs respectively (Table 2). The latter explores how beneficial both discrete LDA and continued LDA entries are to the prediction (Table 3).

**Table 2:** Performance of GRUF in comprehensive scenarios in terms of AUC

Scenario	Binary	Discrete	Continued
T1 (10CV)	0.8916	0.8900	0.9148
T2 (10CV)	0.7505	0.7412	0.8176
T3 (10CV)	0.8487	0.8361	0.8060
T4 (10×10 CV)	0.6080	0.6070	0.6078

**Table 3:** Performance of GRUF in comprehensive scenarios in terms of Correlation

Scenario	Binary	Discrete	Continued
T1 (10CV)	0.1525	0.4012	*0.5709*
T2 (10CV)	0.1498	0.1774	*0.2230*
T3 (10CV)	0.1206	0.1515	*0.3151*
T4 (10×10 CV)	0.1463	0.1541	*0.1583*

The italic entries denote the best

In the first investigation, the values of AUC show that: (1) T1 is the easiest task while T4 is the hardest task across the other three kinds of associations; (2) GRUF shows similar results in binary, discrete and continued prediction over four predicting scenarios.

In the second investigation, the correlation between the predicted confidence scores and the number of RBPs involved in LDAs shows that: (1) continued prediction achieves the best, discrete prediction obtains the moderate, and binary prediction generates the worst results; (2) GRUF usually achieves the best performance in T1 and the worst performance in T4.

Consequently, we may draw the following conclusions: (1) T1 is the easiest task of LDA prediction T2 and T3 are the moderate tasks, and T4 is the hardest task over binary, discrete and continued LDAs in terms of both AUC and Correlation; (2) when utilizing discrete prediction and continued prediction, GRUF has the similar ability to separate LDA from non-LDA; (3) More importantly, GRUF shows its power to capture the cues to the underlying mechanisms of LDA because their correlation between the number of RBPs and the predicted confidence scores of being potential LDAs are higher than that of binary prediction. The last two points enable GRUF to reveal the implications between lncRNA and pathology.

In addition, considering GRUF achieves the most confident prediction in T1, we performed a novel prediction for it to find potential LDAs among DB3 (Table 4). The predicted lncRNA-disease pairs having high confidence scores of being potential associations are ranked. Top-5 out of them were selected to be validated by checking medical literature and LncRNADisease [30] and three among top-5 were validated. The result shows that our approach is able to dig out novel lncRNA-disease associations.

**Table 4:** The validation of potential lncRNA-disease associations in novel prediction

Rank	lncRNA	Disease	Validation
1	DLX6-AS1	breast neoplasms, male	N/A
2	H19	breast neoplasms, male	[32], DB
3	CDKN2B-AS1	breast neoplasms, male	DB
4	DLX6-AS1	musculoskeletal abnormalities	N/A
5	7SK	liver neoplasms, experimental	[33]

DB- LncRNADisease;

N/A- no finding in medical literatures or LncRNADisease

## Conclusions

Existing computational approaches only focus on predicting associations between lncRNAs with known disease association and diseases with known association with some lncRNAs. An open question is whether we can predict association for lncRNAs without known disease association and/or diseases with no known association with any lncRNAs. In addition, current computational approaches only work in the case of binary lncRNA-disease associations (LDA), which cannot reflect and reveal many biological facts or knowledge, such as the number of proteins involved in lncRNA-disease associations and how strong LDAs are. To address abovementioned issues, we have proposed a unified inference approach based on graph regression, GRUF. This proposed GRUF is able to work for four distinct predicting tasks, in particular, for lncRNAs and diseases without any known association. More importantly, it is able to not only perform the prediction of binary LDA but also for both discrete and continued LDAs, which helps revealing the implications between lncRNA and pathology. Experiments on real datasets demonstrate the superiority and effectiveness of our approach. As a remark, we want to emphasize that the results of our approach may be affected by the quality of the dataset and also the expression level of a particular lncRNA. For example, for those lncRNAs with low expression level, it may be difficult for our method or any existing methods to accurately predict its association. For further research, how to tackle these difficult cases would be a challenging problem. Also, we plan to include more disease-related knowledge to improve the accuracy of prediction.

## Abbreviations

(GRUF): graph regression-based unified framework
(LDA): The associations between lncRNAs and diseases
(lncRNAs): long non-coding RNAs
AUC: The area under the receiver operating characteristic curve
AUPR: The area under precision-recall curve
CV: Cross-validation
DAG: Directed acyclic graph
DAM: Disease associating matrix
DLFM: Disease latent feature matrix
ICD: International classification of diseases
MeSH: Medical Subject Headings
PLSR: Partial Least-Squares Regression
RAM: RNA associating matrix
RBP: RNA-binding protein
RLFM: RNA latent feature matrix
